# Characterizing cheat meals among a national sample of Canadian adolescents and young adults

**DOI:** 10.1186/s40337-022-00642-6

**Published:** 2022-08-06

**Authors:** Kyle T. Ganson, Mitchell L. Cunningham, Eva Pila, Rachel F. Rodgers, Stuart B. Murray, Jason M. Nagata

**Affiliations:** 1 Factor-Inwentash Faculty of Social Work, University of Toronto, 246 Bloor Street W, Toronto, ON M5S 1V4 Canada; 2 School of Psychology, The University of Sydney, Sydney, NSW Australia; 3 School of Kinesiology, Western University, London, ON Canada; 4 Department of Applied Psychology, Northeastern University, Boston, MA USA; 5 Department of Psychiatric Emergency & Acute Care, Lapeyronie Hospital, Montpellier, France; 6 Department of Psychiatry and the Behavioral Sciences, University of Southern California, Los Angeles, CA USA; 7 Department of Pediatrics, University of California, San Francisco, CA USA

**Keywords:** Cheat meals, Adolescents, Young adults, Eating disorders, Canadians

## Abstract

**Background:**

“Cheat meals”, described as brief eating episodes that depart from established dietary practices to consume prohibited foods, represent a novel and increasingly common eating behavior with particular salience in adolescence and young adulthood. However, knowledge gaps remain regarding the frequency and characterization of foods and calories consumed during cheat meals, and their associations with eating disorder behaviors and psychopathology. Thus, the aims of this study were to delineate engagement in cheat meals among a large, national sample of Canadian adolescents and young adults.

**Methods:**

Participants (N = 2,717) were from the Canadian Study of Adolescent Health Behaviors. Frequencies of engagement in cheat meals, and associated foods and calories consumed, in the past 12 months and 30 days were determined. The associations between engagement in cheat meals and eating disorder behaviors and psychopathology were determined using modified Poisson regression analyses.

**Results:**

Engagement in cheat meals in the past 12 months was highest among men (60.9%) compared to women (53.7%) and transgender/gender non-conforming (TGNC; 52.5%) participants. Cheat meals consisting between 1,000 and 1,499 cal were those most frequently reported among all participants. Mean number of cheat meals in the past 12 months was equivalent to > 1 per week, which was similar to engagement in the past 30 days. Finally, engagement in cheat meals in the past 12 months and 30 days was associated with patterns of eating disorder behaviors and psychopathology among all participants, including binge-eating-related behaviors.

**Conclusions:**

This study further characterized and extended knowledge of cheat meal engagement across genders, aligning with prior research by demonstrating that engagement is associated with greater eating disorder psychopathology.

**Plain english summary:**

Findings from this study add to the growing characterization of the novel behavioral phenomenon of cheat meals. Specifically, over half of adolescents and young adults across all 13 provinces and territories in Canada reported engaging in > 1 cal dense cheat meal per week, over the past 12 months. Despite the normalization and promotion of cheat meal engagement among the general public and unique fitness communities, engagement in this behavior is linked to greater eating disorder behaviors and psychopathology, including binge-eating episodes. Our findings emphasize the need for further research, public awareness, and clinical interventions aimed at addressing this potentially harmful eating behavior.

**Supplementary Information:**

The online version contains supplementary material available at 10.1186/s40337-022-00642-6.

## Introduction

Muscularity-oriented eating and body-change behaviors have garnered new research and clinical interest given a recent rise in prevalence [1, 2]. Generally, these behaviors can be characterized by potentially dysfunctional body change strategies aimed at increasing lean muscle mass and reducing body fat, and can include excessive exercising and weight lifting, protein overconsumption, “bulk” and “cut” food intake cycles, intermittent fasting, performance-enhancing substance use, and “cheat meals” [3–6]. While the majority of research in these fields has been devoted to elucidating the prevalence and correlates of behaviors such as performance-enhancing substance use [7–9], considerably less research has focused on specific dietary behaviors that fall under the category of muscularity-oriented eating and body-change behaviors. Recently, there has been a small, but growing literature characterizing the newer eating phenomenon of “cheat meals”, however, there remains major gaps in current understanding of the relationship between cheat meals and eating disorder psychopathology.

Cheat meals have previously been described in the literature as eating episodes that temporarily deviate from one’s established dietary practices (i.e., restrictive and/or restraint) to consume prohibited foods momentarily, only to return back to the previous dietary practices (i.e., a “cheating” deviation from regular rigid eating practices) [10]. Within the context of muscularity-oriented eating and body-change behaviors, cheat meal engagement, and the often-times accompaniment of high calorie consumption, is intended (at least by some within the fitness culture) to manipulate the body to using dietary fats as energy and enhance body fat reduction without negatively impacting muscle development [10]. To further characterize cheat meals, prior research explored the hashtag #cheatmeals to analyze the themes present in user posted images on Instagram [11]. Findings revealed that engagement in cheat meals was often depicted by a large amount of food representing a very high caloric density estimated at approximately 3000 cal (e.g., large pizzas, tubs of ice cream, very large portions of pasta). Relatedly, the study further situated cheat meals as a primary and sanctioned eating behavior within the context and community of individuals attempting to achieve the muscular ideal [11]. To highlight the continued popularity of cheat meals, a brief investigation of the hashtag #cheatmeal on Instagram populated over 4.2 million images and videos as of March 25, 2022, underscoring the need for future research on this eating behavior.

Given that cheat meals are in part intended for the pursuit of a socially prescribed, and often unattainable, body ideal (i.e., overly lean and muscular), it is relevant to explore the relationships between engagement in cheat meals and eating disorder behaviors and psychopathology. Despite cheat meals being viewed more positively in specific social situations, prior research has acknowledged overlaps between cheat meals and binge-eating episodes [10, 11] underscoring potential psychopathological characteristics of cheat meals. To date, only one study (to our knowledge) has explored the relationships between cheat meals and eating disorder behaviors and psychopathology using a modest sample (N = 248) of undergraduate kinesiology students in Canada [12]. Findings from this study showed that engagement in cheat meals was more commonly unplanned (85%) compared to planned (66%) and used for a variety of purposes. This included cheat meals to help control food cravings, which may be seen as a mechanism to avoid binge-eating, to supplement fitness and exercise activities, and to improve metabolism [12]. Additionally, the study found that engagement in cheat meals was associated with higher levels of overall eating disorder psychopathology, measured using the Eating Disorder Examination Questionnaire (EDE-Q) Global Score [13], and objective binge-eating among men but not women [12]. These findings are important in describing patterns of engagement in cheat meals; however, are limited given the small, convenience sample, thus limiting generalizability, as well as only focusing on men and women. In addition, the study did not explore areas such as estimated calories consumed during cheat meals and types of foods consumed. This is particularly important given that cheat meals may be defined differently from person-to-person. Thus, gathering such information will provide helpful data to conceptualize and describe this eating behavior.

To address gaps in the literature on cheat meals, this study has the following three aims: 1) to describe the frequency of engagement in cheat meals over the past 12 months and 30 days; 2) to characterize the caloric composition and dietary preferences of foods consumed during cheat meals; and 3) to determine the associations between cheat meals and eating disorder behaviors and psychopathology. Given prior research [11, 12] and the presence of this eating behavior on social media applications, it was hypothesized that engagement in cheat meals would occur at high prevalence and would be associated with consuming high calorie foods and greater eating disorder behaviors and psychopathology.

## Methods

The Canadian Study of Adolescent Health Behaviors was a national study of Canadian adolescents and young adults that surveyed participants on a range of eating, exercise, and social and behavioral health behaviors. Participants (N = 2,717) were recruited online using Instagram and Snapchat advertisements from November 2021 through December 2021 (see Additional file 1: Supplement Table 1 for description of social media recruitment). Survey advertisements were restricted to Canada (location), both sexes (males and females; note that both social media applications do not provide gender options nor intersex), and ages 16–30 years. No other social media advertisement targeting techniques were used to allow advertisements to be displayed to any individual meeting these criteria. This method of participant recruitment was utilized given that over 87% of Canadians ages 15–35 years engage with social media applications and platforms [14]. In order to participate in the study, individuals must have been between the ages of 16–30 years at the time of study, currently living in Canada, and read English. There were no other inclusion or exclusion criteria. Participants completed an online survey via Qualtrics and were able to enter to win one of two Apple iPads or one of 20 $25 Starbucks gift cards as compensation for their participation. Participants were informed that the study focused on social and behavioral health behaviors, including disordered eating behaviors, muscle-building behaviors, and body image, as well as the social health, of adolescents and young adults aged 16–30 years old in Canada. The goal of the study was to develop research-informed recommendations for health care, public health, and policymaking professionals to protect the health and well-being of Canadian young people. Informed consent was obtained from all participants and ethics approval was obtained from the Health Sciences Research Ethics Board at the University of Toronto (#41707). 

### Measures

*Engagement in cheat meals* was determined using two questions that assessed engagement in the past 12 months and past 30 days (“yes” or “no”). No specific definition of cheat meals was provided to participants given the novel and evolving nature of this eating behavior. If a participant reported they engaged in a cheat meal in the past 12 months or past 30 days, subsequent questions were revealed to characterize their engagement. *Typically consumed foods during cheat meals* was assessed using the question, “Over the [*past 12 months/past 30 days*], what types of foods did you *typically* consume during a "cheat meal"? Select all that apply.” Response options included, “Calorically dense foods (e.g., burgers, fries, pizza, mac and cheese);” “Dairy foods (e.g., ice cream, cheese, milk);” “High carbohydrate foods (e.g., pastas, breads, bagels);” “High fat foods (e.g., nut butters, nuts/trail mix, avocados);” “High protein foods (e.g., chicken, meat, fish);” “Salty foods (e.g., potato chips, popcorn);” “Sweet foods (e.g., candy, cookies, desserts);” and “Other (please specify).” *Estimated number of calories consumed during typical cheat meals* was assessed using the question, “Over the [*past 12 months/past 30 days*], roughly how many calories do you think you consumed during a *typical* "cheat meal"? Response options included, “Less than 500 cal;” “500 to 999 cal;” “1,000 to 1,499 cal;” “1,500 to 1,999 cal;” “2,000 to 2,499 cal;” “2,500 to 2,999 cal;” “3,000 or more calories;” and “Other (please specify).” Lastly, *estimated number of times engaged in cheat meals* was assessed using the question, “Over the [*past 12 months/past 30 day*], roughly how many *times* did you engage in a "cheat meal"? Response options ranged from 1–365 (past 12 months) to 1–50 (past 30 days).

*Eating disorder psychopathology* was determined using the Eating Disorder Examination Questionnaire (EDE-Q) 6.0 Global Score [13]. The EDE-Q Global Score was determined from the mean score of four subscales (i.e., Dietary Restraint, Eating Concerns, Weight Concerns, and Shape Concerns). Cronbach's α for the Global Score was excellent for men (0.92), women (0.96), and (0.95) for TGNC participants.

*Disordered eating behaviors* were measured using the single-item measures within the EDE-Q [13]. This included the presence (≥ 1) or absence (0) of overeating, loss of control while eating, binge-eating, and compensatory behaviors for the purposes of altering body shape or weight, including vomiting, laxative use, compulsive exercise, and fasting, all over the course of the past 28 days at the time of study. These items were dichotomized given the sample is community-based and the data were not normally distributed, aligning with prior research [15–20].

Demographic and control variables included self-reported race/ethnicity (White or Caucasian; Black; Latino/a; East/Southeast Asian; South Asian; Middle Eastern; Indigenous; other race/ethnicity; and multi-racial), sexual identity (heterosexual; gay/lesbian; bisexual; and queer, questioning, or other), highest education completed (high school diploma or less; college or undergraduate degree; master’s degree or higher; other), weight perception (very underweight; slightly underweight; about the right weight; slightly overweight; very overweight) and current weight change behaviors (lose weight; stay the same weight; gain weight; not doing anything about weight). Sex at birth (male; female) and current gender identity (female; male; trans male/trans man; trans female/trans woman; genderqueer/gender non-conforming; gender non-binary; other) were also assessed. A three-category gender variable (cisgender woman; cisgender man; transgender/gender non-conforming individual) was created for analytic purposes.

### Analysis plan

Descriptive statistics using means (M) and standard deviations (SD), and frequencies (percentages) were computed among the sample and gender differences were assessed using a series of one-way ANOVAs and chi-square tests for continuous and categorical variables, respectively. Unadjusted frequencies of typically consumed foods during cheat meals and estimated number of calories consumed during typical cheat meals and means and SD of the estimated number of times engaged in cheat meals, were estimated. Multiple modified Poisson regression models using robust error variance [21] were estimated and adjusted rate ratios (ARR) and 95% confidence intervals (CI) were used to determine the association between the presence or absence of engagement in cheat meals (both past 12 months and past 30 days) and the eating disorder behaviors and EDE-Q Global Score, while adjusting for the demographic and control variables. All analyses were stratified by gender given differing levels of engagement and purpose of muscularity-oriented eating and body-change behaviors, including cheat meals, across genders [10, 12, 22, 23]. Statistical significance was defined as two-sided *p* < 0.05 using Stata 17.

## Results

Overall, the sample was demographically diverse and consisted of 53.5% women and 62.4% White participants (Table 1). Engagement in cheat meals in the past 12 months was higher among men (60.9%) compared to women (53.7%) and TGNC (52.5%) participants. Among those who reported engagement in cheat meals in the past 12 months, 87.8% of men, 82.5% of women, and 73.9% of TGNC participants reported engaging in a cheat meal in the past 30 days. The EDE-Q Global Score was highest among TGNC participants (M = 2.8, SD = 1.6) followed by women (M = 2.6, SD = 1.5), and men (M = 1.5, SD = 1.2). Similarly, engagement in six of the seven eating disorder behaviors was significantly higher among TGNC participants compared to men and women. TGNC and men reported similar engagement in overeating, which was higher than that of women.

**Table 1:** Sample characteristics of 2,717 Canadian participants ages 16–30 years old

	Women (n = 1,476)	Men (n = 1,060)	TGNC (n = 181)		
	M (SD) / %	M (SD) / %	M (SD) / %	p^a^	*F*^b^ / V^c^
Age	23.1 (3.9)	22.8 (3.9)	21.8 (3.9)	< .001	8.21
Race/ethnicity				< .001	.10
White	65.2	57.5	70.6		
Black	3.2	3.1	2.8		
Latino	1.9	3.2	0.6		
East Asian	9.3	10.8	9.6		
South Asian	5.3	10.2	2.8		
Middle Eastern	1.9	3.2	1.1		
Indigenous	1.5	1.0	1.1		
Other	1.3	1.6	0.0		
Multi-racial	10.4	9.3	11.3		
Sexual identity				< .001	.35
Heterosexual	57.3	70.2	5.6		
Gay/lesbian	2.4	14.4	16.4		
Bisexual	23.8	8.7	24.9		
Queer, questioning, or other	16.5	6.7	53.1		
Highest completed education				.004	.06
High school diploma or less	41.7	45.0	57.1		
College or undergraduate degree	43.8	43.3	33.3		
Master’s degree or higher	12.9	10.7	7.9		
Other	1.6	1.0	1.7		
Weight perception				< .001	.09
Very underweight	1.2	1.8	1.7		
Slightly underweight	8.8	14.8	10.1		
About the right weight	48.0	50.7	47.5		
Slightly overweight	32.8	27.6	32.4		
Very overweight	9.2	4.9	8.4		
Current weight change behavior				< .001	.32
Lose weight	57.2	32.3	52.5		
Stay the same weight	17.9	14.5	12.3		
Gain weight	7.1	44.3	10.1		
Not doing anything about weight	17.9	8.9	25.1		
Cheat meals, past 12 months	53.7	60.9	52.5	.001	.07
Cheat meals, past 30 days^d^	82.5	87.8	73.9	< .001	.11
Any overeating, past 28 days	63.0	71.4	71.4	< .001	.09
Any loss of control while eating, past 28 days	54.8	32.1	64.3	< .001	.24
Any binge-eating, past 28 days	49.6	29.1	60.7	< .001	.22
Any vomiting, past 28 days	13.2	6.5	30.8	< .001	.20
Any laxative use, past 28 days	9.4	3.9	27.7	< .001	.22
Any compulsive exercise, past 28 days	50.0	42.2	55.8	< .001	.09
Any fasting, past 28 days	44.8	27.6	62.5	< .001	.22
EDE-Q global score	2.6 (1.5)	1.5 (1.2)	2.8 (1.6)	< .001	27.09

Women and men were defined as those who identified as cisgender (i.e., their sex at birth aligned with their current gender identity)

*M* Mean, *SD* Standard deviation, *TGNC* Transgender/Gender Non-Conforming, *EDE-Q* Eating Disorder Examination Questionnaire

^a^Differences between genders determined using chi-square tests for categorial variables and one-way ANOVAs for continuous variables

^b^*F* statistic from one-way ANOVA’s for continuous variables

^c^Effect size determined using Cramer’s V for categorial variables

^d^Only asked of those who reported “yes” to cheat meals in the past 12 months

**Fig. 1:**
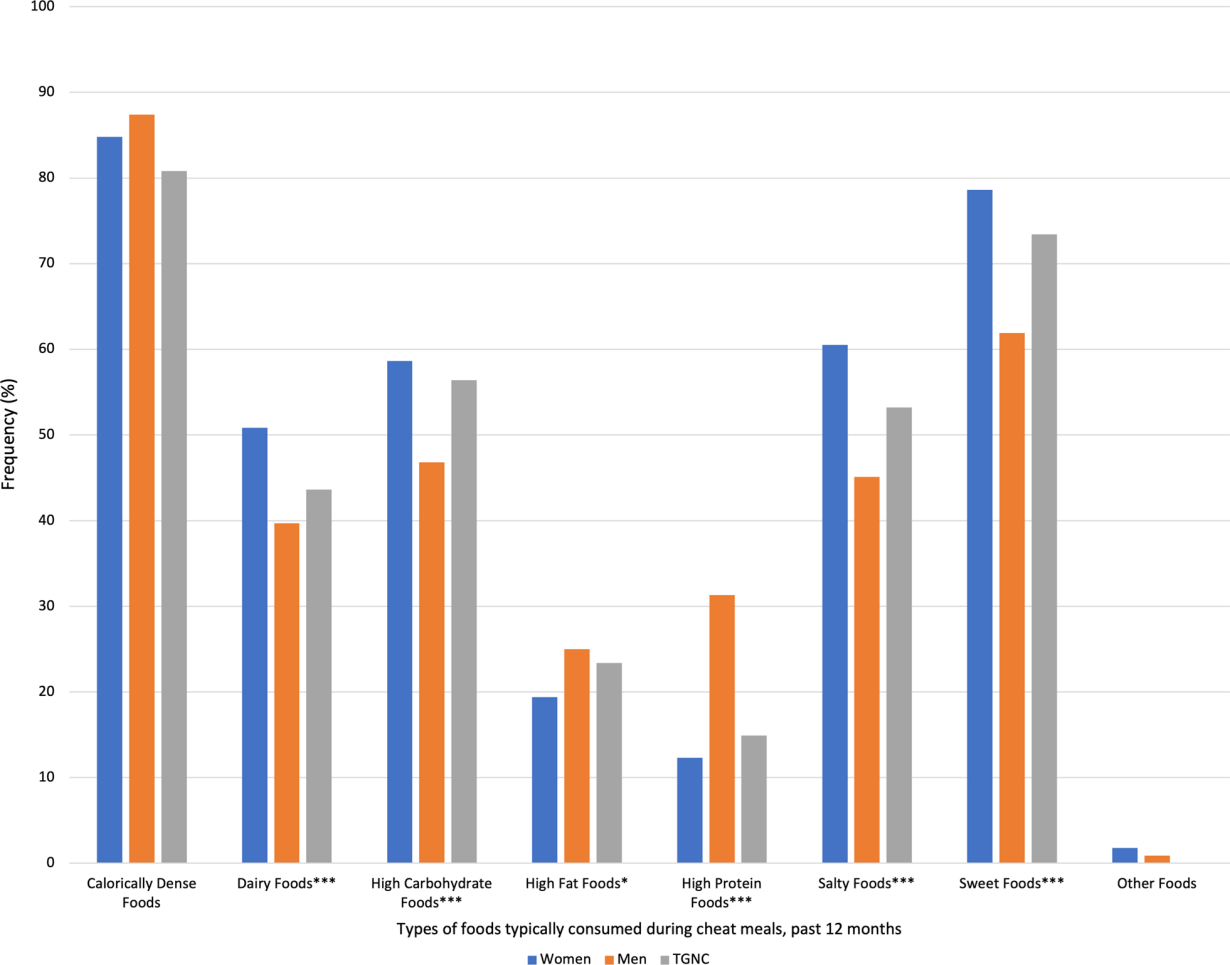
Frequency of typically consumed foods during cheat meals in the past 12 months by gender. *Note*: Frequencies are among those who reported “yes” to a cheat meal in the past 12 months. Differences between foods and gender determined using chi-square tests (*p < .05, ***p < .001). See Results section for Cramer’s V. TGNC = Transgender/gender non-conforming

**Fig. 2:**
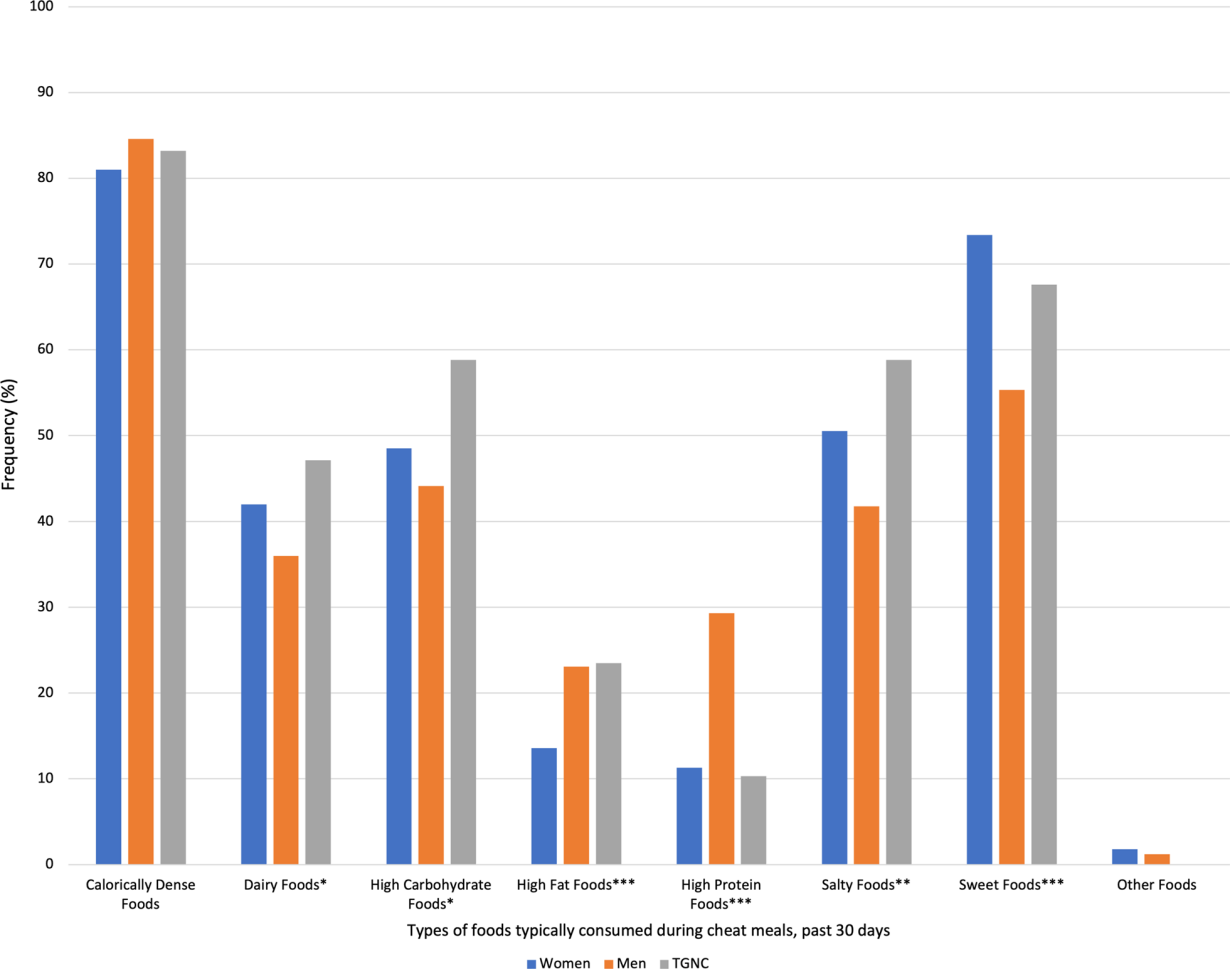
Frequency of typically consumed foods during cheat meals in the past 30 days by gender. *Note*: Frequencies are among those who reported “yes” to a cheat meal in the past 30 days. Differences between foods and gender determined using chi-square tests (*p < .05, ***p < .001). See Results section for Cramer’s V. TGNC = Transgender/gender non-conforming

Regarding the foods typically consumed during cheat meals over the past 12 months and past 30 days (Figs. 1 and 2), descriptively, calorie dense foods were highest across the entire sample. However, significant differences emerged between genders in regards to foods consumed in the past 12 months, including dairy foods (women: 50.8%, men: 39.7%, TGNC: 43.6%, Cramer’s V = 0.11), high carbohydrate foods (women: 58.6%, men: 46.8%, TGNC: 56.4%, Cramer’s V = 0.11), high fat foods (women: 19.4%, men: 25.0%, TGNC: 23.4%, Cramer’s V = 0.07), high protein foods (women: 12.3%, men: 31.3%, TGNC: 14.9%, Cramer’s V = 0.23), salty foods (women: 60.5%, men: 45.1%, TGNC: 53.2%, Cramer’s V = 0.15), and sweet foods (women: 78.6%, men: 61.9%, TGNC: 73.4%, Cramer’s V = 0.18).

This pattern remained largely the same for foods consumed in the past 30 days, including dairy foods (women: 42.0%, men: 36.0%, TGNC: 47.1%, Cramer’s V = 0.07), high carbohydrate foods (women: 48.5%, men: 44.1%, TGNC: 58.8%, Cramer’s V = 0.07), high fat foods (women: 13.6%, men: 23.1%, TGNC: 23.5%, Cramer’s V = 0.12), high protein foods (women: 11.3%, men: 29.3%, TGNC: 10.3%, Cramer’s V = 0.23), salty foods (women: 50.5%, men: 41.8%, TGNC: 58.8%, Cramer’s V = 0.10), and sweet foods (women: 73.4%, men: 55.3%, TGNC: 67.6%, Cramer’s V = 0.18).

**Fig. 3:**
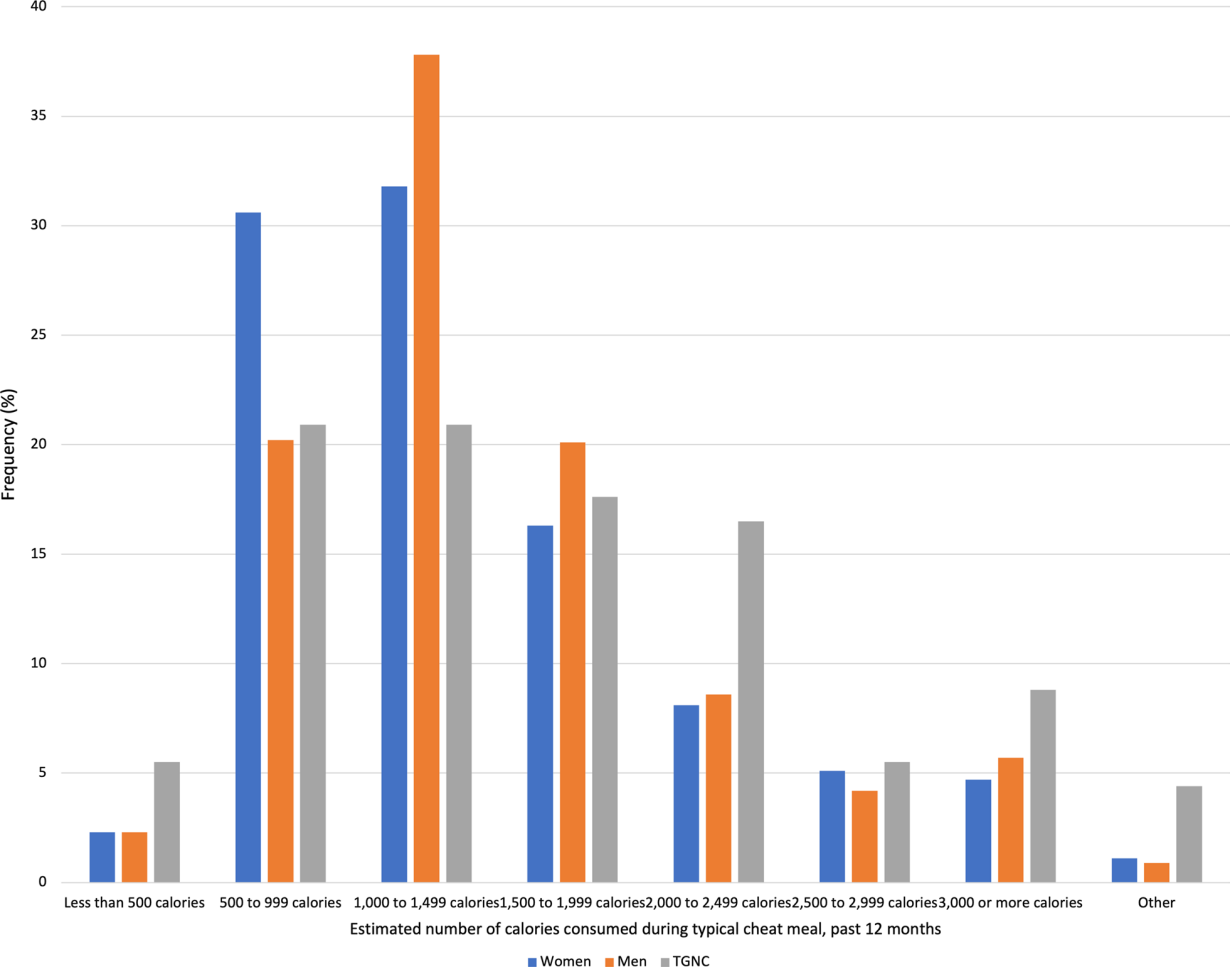
Frequency of estimated number of calories consumed during typical cheat meals in the past 12 months by gender. *Note*: Frequencies are among those who reported “yes” to a cheat meal in the past 12 months. Statistically significant (Cramer’s V 0.13, *p* < .001) differences between genders determined using chi-square test. TGNC = Transgender/gender non-conforming

**Fig. 4:**
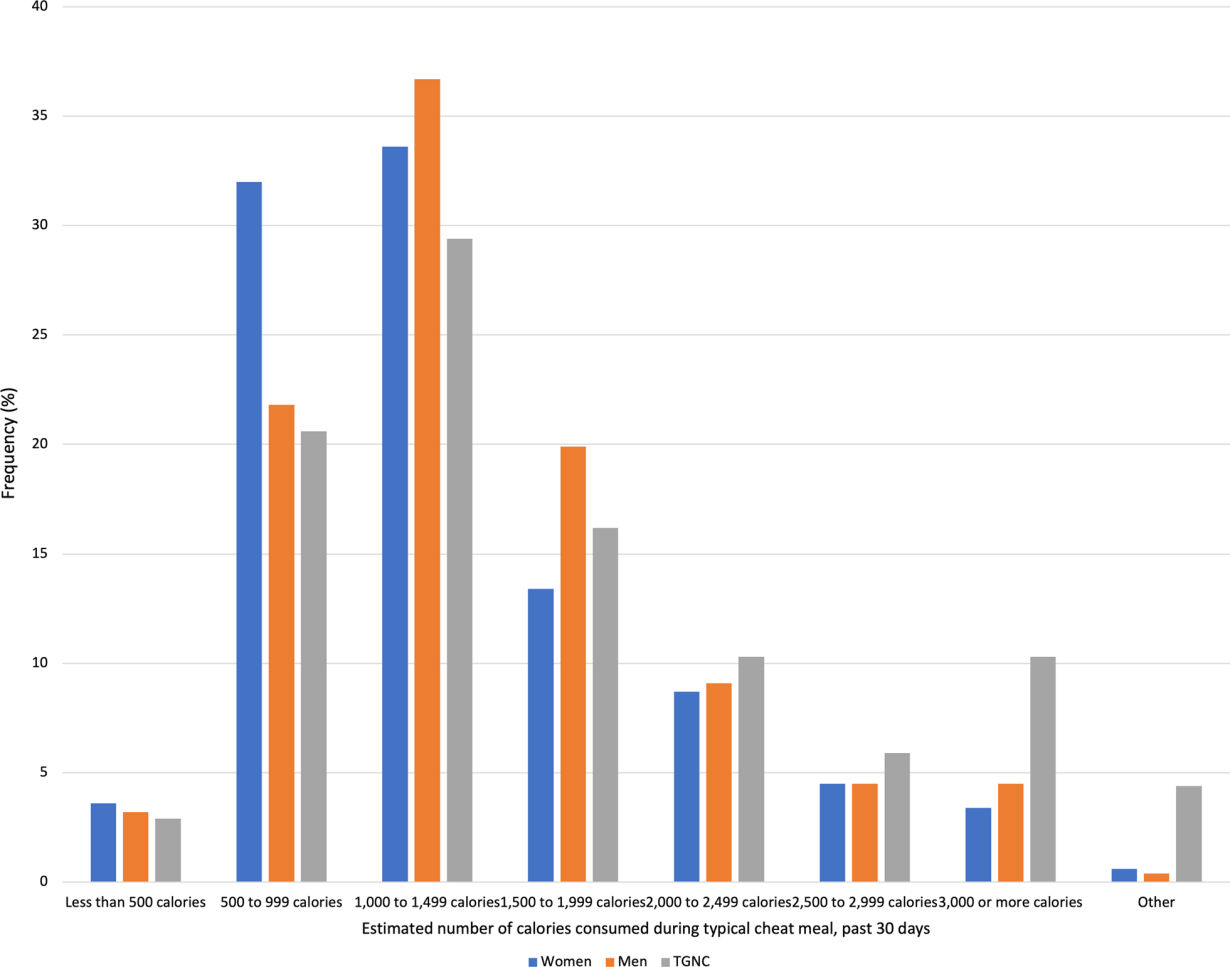
Frequency of estimated number of calories consumed during typical cheat meals in the past 30 days by gender. *Note*: Frequencies are among those who reported “yes” to a cheat meal in the past 30 days. Statistically significant (Cramer’s V 0.13, *p* < .001) differences between genders determined using chi-square test. TGNC = Transgender/gender non-conforming

Regarding estimated number of calories consumed during typical cheat meals in the past 12 months and past 30 days (Figs. 3 and 4), significant differences between genders in calories consumed emerged. In the past 12 months, both men (37.8%) and women (31.8%) reported consuming between 1,000 and 1,499 cal, while TGNC participants reported consuming between 500 and 999 cal (20.9%) and 1,000 and 1,499 cal (20.9%) during a typical cheat meal. In the past 30 days, men (36.7%), women (33.6%), and TGNC participants (29.4%) reported consuming between 1,000 and 1,499 cal during a typical cheat meal.

**Fig. 5:**
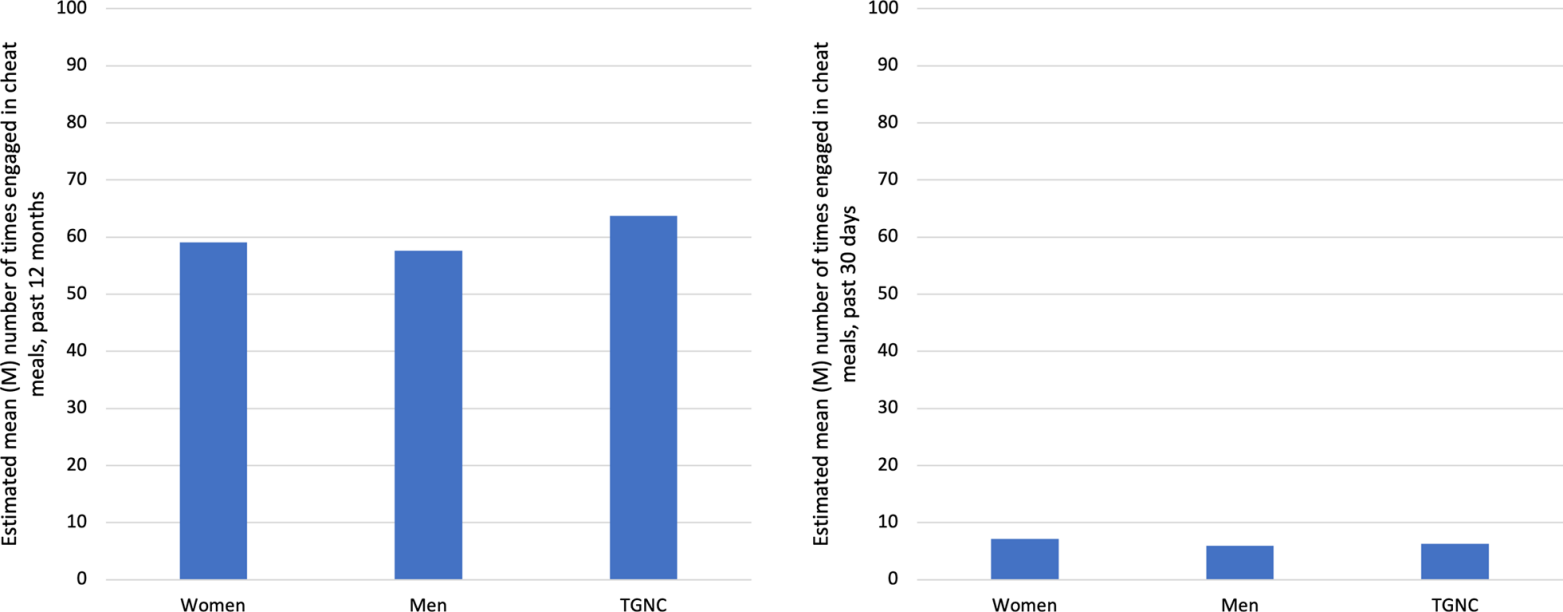
Estimated mean number of times engaged in cheat meals in the past 12 months and past 30 days by gender. *Note*: Means are among those who reported “yes” to a cheat meal in the past 12 months and past 30 days. No statistically significant differences in mean between genders were found using one-way ANOVA for past 12-month cheat meal engagement. Statistically significant (*F* 5.28, *p* < .01) differences in means between genders were found using one-way ANOVA for past 30-day cheat meal engagement. TGNC = Transgender/gender non-conforming

Regarding estimated mean number of times participants engaged in cheat meals (Fig. 5), there were no significant differences in mean number of times in the past 12 months across genders. Conversely, in the past 30 days, women (M = 7.1, SD = 7.0) reported significantly higher number of cheat meals compared to men (M = 5.9, SD = 6.1) and TGNC participants (M = 6.2, SD = 4.3).

Finally, engagement in cheat meals was associated with greater eating disorder behaviors and greater eating disorder psychopathology (Table 2). Regarding eating disorder behaviors, among women, engagement in cheat meals in the past 12 months was associated with all seven eating disorder behaviors, while engagement in cheat meals in the past 30 days was associated with six eating disorder behaviors. Among men, engagement in cheat meals in the past 12 months and 30 days were associated with any binge-eating, any compulsive exercise, and any fasting. Among TGNC participants, engagement in cheat meals in the past 12 months and 30 days were associated with any overeating and any binge-eating, while engagement in cheat meals in the past 30 days was also associated with any loss of control while eating.

**Table 2:** Associations between engagement in cheat meals in the past 12 months and 30 days and eating disorder behaviors and psychopathology

	Cheat meals, past 12 months		Cheat meal, past 30 days^a^	
	ARR^b^ (95% CI)	*p*	ARR^b^ (95% CI)	*p*
*Women*				
Any overeating, past 28 days	**1.29 (1.12–1.48)**	**< .001**	**1.27 (1.11–1.46)**	**< .001**
Any loss of control while eating, past 28 days	**1.44 (1.24–1.69)**	**< .001**	**1.38 (1.19–1.59)**	**< .001**
Any binge-eating, past 28 days	**1.47 (1.25–1.73)**	**< .001**	**1.39 (1.19–1.62)**	**< .001**
Any vomiting, past 28 days	**1.59 (1.14–2.21)**	**.006**	**1.40 (1.03–1.89)**	**.030**
Any laxative use, past 28 days	**1.77 (1.20–2.62)**	**.004**	1.41 (0.98–2.02)	.061
Any compulsive exercise, past 28 days	**1.33 (1.13–1.55)**	**< .001**	**1.28 (1.10–1.49)**	**.001**
Any fasting, past 28 days	**1.66 (1.39–1.98)**	**< .001**	**1.57 (1.33–1.85)**	**< .001**
EDE-Q Global Score	**1.31 (1.22–1.42)**	**< .001**	**1.27 (1.19–1.36)**	**< .001**
*Men*				
Any overeating, past 28 days	1.16 (0.99–1.35)	.067	1.15 (0.99–1.34)	.058
Any loss of control while eating, past 28 days	1.20 (0.94–1.52)	.133	1.22 (0.97–1.53)	.086
Any binge-eating, past 28 days	**1.34 (1.02–1.70)**	**.034**	**1.32 (1.03–1.67)**	**.025**
Any vomiting, past 28 days	0.80 (0.48–1.34)	.398	0.69 (0.41–1.14)	.149
Any laxative use, past 28 days	1.17 (0.58–2.36)	.664	0.72 (0.37–1.39)	.326
Any compulsive exercise, past 28 days	**1.35 (1.10–1.67)**	**.005**	**1.31 (1.07–1.60)**	**.007**
Any fasting, past 28 days	**1.46 (1.12–1.92)**	**.006**	**1.29 (1.01–1.66)**	**.042**
EDE-Q Global Score	**1.35 (1.21–1.51)**	**< .001**	**1.33 (1.20–1.48)**	**< .001**
*TGNC participants*				
Any overeating, past 28 days	**1.82 (1.18–2.81)**	**.007**	**1.89 (1.24–2.89)**	**.003**
Any loss of control while eating, past 28 days	1.54 (0.97–2.44)	.063	**1.63 (1.05–2.54)**	**.031**
Any binge-eating, past 28 days	**1.87 (1.14–3.06)**	**.013**	**1.99 (1.24–3.19)**	**.004**
Any vomiting, past 28 days	1.34 (0.53–3.41)	.534	0.67 (0.26–1.70)	.399
Any laxative use, past 28 days	1.53 (0.51–4.61)	.452	1.87 (0.64–5.44)	.249
Any compulsive exercise, past 28 days	1.31 (0.79–2.17)	.293	0.95 (0.58–1.55)	.833
Any fasting, past 28 days	1.08 (0.69–1.68)	.746	1.13 (0.73–1.76)	.580
EDE-Q Global Score	**1.24 (1.01–1.51)**	**.037**	1.15 (0.94–1.40)	.165

Each cell represents the abbreviated outputs of modified Poisson regression models with robust error variance

Boldface indicates statistical significance (*p* < .05)

*ARR* Adjusted rate ratio, *CI* Confidence interval, *TGNC* Transgender/gender non-conforming, *EDE-Q* Eating Disorder Examination Questionnaire

^a^Includes all those who did (1) and didn’t (0) report engagement in cheat meals in the past 30 days

^b^Adjusted for race/ethnicity, sexual identity, highest education completed, weight perception, and current weight change behaviors

Regarding eating disorder psychopathology (Table 2), engagement in cheat meals in the past 12 months was associated with greater EDE-Q Global Score among men (ARR = 1.35, 95% CI 1.21–1.51), women (ARR = 1.31, 95% CI 1.22–1.42), and TGNC participants (ARR = 1.24, 95% CI 1.01–1.51), while adjusting for demographic and confounding variables. Similarly, engagement in cheat meal in the past 30 days was associated with greater eating disorder psychopathology among men (ARR = 1.33, 95% CI 1.20–1.48) and women (ARR = 1.27, 95% CI 1.19–1.36), while adjusting for demographic and confounding variables.

## Discussion

The results from this study underscore the common nature of engagement in cheat meals among a large, national sample of adolescents and young adults in Canada. Specifically, over half of men, women, and TGNC participants reported engaging in at least one cheat meal in the past 12 months. Cheat meals occurred at lower frequency compared to prior research [12], which is likely explained by the larger and more diverse sample of participants in the current study. Conversely, the findings from this study are consistent with prior research supporting the association between engagement in cheat meals and eating disorder behaviors and psychopathology [12], including binge-eating-related behaviors, highlighting the potentially problematic nature of this eating behavior and its relevance to disordered eating.

Several additional unique findings should be noted. While the frequency of consuming calorie dense foods was similar across all participants, significant differences emerged between genders regarding foods typically consumed during cheat meals. These included the greater frequency of men reporting high protein foods in comparison to women and TGNC participants. Given that cheat meals have been conceptualized and promoted within the muscle-building and fitness community [10], this finding supports the theory that men in this study may be strategically using cheats meals to catalyze muscle growth through large consumptions of dietary protein. Conversely, the greater frequency of consuming dairy foods, salty foods, and sweet foods among women compared to men may be indicative of women engaging in cheat meals as a means to prevent or curtail binge-eating episodes, or to alleviate cravings for otherwise restricted foods. Furthermore, research has shown that women often prefer sweets such as chocolates and ice cream, while men prefer hearty meals such as steak, during binge-eating episodes [24]. Conversely, there was less of a pattern of cheat meal food preferences among TGNC individuals. However, TGNC participants overall reported a greater number of calories consumed during a typical cheat meal. This included approximately 10% of TGNC participants who reported consuming 3,000 cal or more during a typical cheat meal in the past 12 months and past 30 days. This finding may be evidence that typical cheat meals align more with binge-eating episodes among TGNC participants compared to men and women. This is supported by the findings showing greater effect sizes for the relationship between cheat meals and binge-eating among TGNC participants compared to men and women. Finally, while men, women, and TGNC participants reported approximately the same number of cheat meals in the past 12 months, women reported a slightly higher mean number of times in the past 30 days compared to men and TGNC participants. In fact, women reported approximately seven cheat meals within the past 30 days, which is equivalent to nearly two per week. While speculative, this may be indicative of cheat meals serving a different purpose for women compared to men and TGNC participants.

Study findings highlight the link between engagement in cheat meals and eating disorder behaviors and psychopathology. Specifically, women who reported cheat meals in the past 12 months also reported higher rates of all seven eating disorder behaviors under study, including binge-eating, and compensatory behaviors to control one’s shape and weight, such as vomiting, laxative use, compulsive exercise, and fasting. This was consistent for women who engaged in cheat meals in the past 30 days with the exception of laxative use. Among men, engagement in cheat meals in the past 12 months and 30 days were associated with higher rates of binge-eating, and compulsive exercise and fasting to control shape or weight. Finally, among TGNC participants, engagement in cheat meals in the past 12 months and 30 days were associated with episodes of overeating and binge-eating. Finally, with the exception of cheat meal engagement in the past 30 days among TGNC participants, engagement in cheat meals in the past 12 months and 30 days were significantly associated with greater eating disorder psychopathology as measured by the EDE-Q Global Score [13], with men having a marginally higher effect size compared to women and TGNC participants. Taken together, these findings highlight unique patterns of eating disorder behaviors associated with cheat meals, including specific behavioral differences across genders, further underscoring the potentially problematic nature of this eating behavior.

While study findings have significant implications for researchers and clinical professionals, several limitations should be noted prior to providing implications. First, participants were sampled using a nonprobability method, therefore, the findings cannot be generalized to the entire Canadian population and may have introduced sampling bias (i.e., selection bias). Additionally, response rates in relation to number of social media advertisement impressions and reach were low. However, response rates were comparable with prior research using social media advertisements [25, 26]. Despite this limitation, participants represented all 13 provinces and territories in Canada and were gathered via two commonly used social media outlets in Canada [14] without the use of specific adverting targeting features. Future research should focus on obtaining a nationally representative sample of Canadian adolescents and young adults to extend the findings. Second, all items are based on self-report and relied on participants to estimate their engagement in cheat meals, which may introduce reporting, recall, and social desirability bias, particularly related to the 12-month recall of the frequency, foods, and volume of cheat meals. Future research should consider ways to measure cheat meal engagement objectively, such as ecological momentary assessment, daily dairy studies, or interviews to measure and describe the psychopathological aspects of cheat meals more comprehensively. Additionally, while we strategically did not provide a definition of cheat meals to participants to capture a wide array of interpretations of this behavioral phenomenon, there is the possibility that interpretation of cheat meals varied across participants, thus potentially influencing participant responses to the question. However, given the colloquial popularity and interest in cheat meals within the general population and fitness communities, significant deviations of interpretation are unlikely. Third, the data are cross-sectional, preventing us from drawing casual inferences, specifically regarding assessing the association between cheat meal engagement and eating disorder behaviors and psychopathology. Future longitudinal research is needed to describe whether cheat meals are prospectively associated with eating disorder behaviors and psychopathology or may in fact reduce it as intended by many who believe that cheat meals will reduce cravings and binge-eating. Further, while the EDE-Q is commonly used in eating disorders research, the questionnaire may not capture unique eating disorder cognitions and behaviors particularly among men and TGNC individuals, as well as regarding muscularity-oriented behaviors. Notable study strengths include the large, national, and diverse sample, as well as the use of a variety of items to describe individual engagement in cheat meals.

Considering these strengths and limitations, the study findings have important implications for researchers and clinical professionals. Researchers should consider the varying ways in which men, women, and TGNC individuals engage in cheat meals to inform future research. Specifically, the findings add to the growing conceptualization of this eating behavior as the consumption of calorie dense foods, often salty or sweet, occurring at least one time per week. Indeed, this information can be used to inform future investigations. Additionally, qualitative research will be key in further delineating the lived experiences from individuals who engage in cheat meals, as well as more specifically define the purpose of such behavior (i.e., the use of cheat meals for muscularity-oriented purposes and/or to curtail or avoid binge-eating episodes). This future research is crucial given the links between cheat meals and distinct maladaptive eating behaviors and general eating disorder psychopathology found in this study. Clinical professionals should be aware of the common occurrence of cheat meals among adolescents and young adults in this sample, as well as the sanctioned nature of these behaviors in fitness communities [10] and on social media [11]. Screening for cheat meals, and subsequent eating disorder behaviors and psychopathology, is recommended given the high frequency of this behavior found in this study. Education and appropriate treatment and referrals may also be indicated.

## Conclusion

The findings from this study support and extend the prior, albeit nascent, research on cheat meals, showing that over half of adolescent and young adult participants from across Canada reported engaging in cheat meals in the past 12 months, and that engagement in cheat meals was associated with greater engagement in eating disorder behaviors and psychopathology. Additionally, men reported typically consuming high protein foods during cheat meals, while women and TGNC participants reported typically consuming sweet foods. Men, women, and TGNC participants reported their cheat meals were calorically dense and were between 1,000 and 1,499 cal. Finally, men, women, and TGNC participants reported engaging in an average of more than one cheat meal per week over the past 12 months and 30 days. These findings further characterize cheat meals within the tradition of muscularity-oriented eating and body-change behaviors and underscore the overlap between this new behavior and eating disorder psychopathology.

## Supplementary Information

**Additional file 1**. Supplement Table 1.

## Data Availability

Data may be made available upon reasonable request.
