# Supplementary material for: Characterizing cheat meals among a national sample of Canadian adolescents and young adults
Source: J Eat Disord. 2022 Aug 6;10:113. doi: 10.1186/s40337-022-00642-6 (PMC9357326; doi:10.1186/s40337-022-00642-6)
Supplement: Supplementary file 1 — Additional file 1. Supplement Table 1. [file 40337_2022_642_MOESM1_ESM.docx]

| Supplement Table 1  Social media advertising results | |
| --- | --- |
| Instagram Ads |  |
| Total Impressions^a^ | 851,259 |
| Total Reach^b^ | 539,235 |
| Total Clicks^c^ | 8,237 |
| Snapchat Ads |  |
| Total Impressions^a^ | 309,298 |
| Total Reach^b^ | 184,802 |
| Total Clicks^c^ | 4,273 |
| Total |  |
| Total Impressions^a^ | 1,160,557 |
| Total Reach | 724,037 |
| Total Clicks^c^ | 12,510 |
| Started Surveys | 2,772 |
| Completed Surveys | 2,729^d^ |
| Response Rates |  |
| 21.8% | Completed surveys from total clicks |
| 0.4% | Completed surveys from total reach |
| 0.2% | Completed surveys from total impressions |
| ^a^Impressions are the number of times ads were seen on screens.  ^b^Reach is the number of people who saw ads at least once.  ^c^Clicks are the number of times ad links were clicked.  ^d^43 individuals did not provide consent to participate. | |
